# High Serum Carbohydrate Antigen (CA) 125 Level Is Associated With Poor Prognosis in Patients With Light-Chain Cardiac Amyloidosis

**DOI:** 10.3389/fcvm.2021.692083

**Published:** 2021-10-28

**Authors:** Muzheng Li, Zhijian Wu, Ilyas Tudahun, Na Liu, Qiuzhen Lin, Jiang Liu, Yingmin Wang, Mingxian Chen, Yaqin Chen, Nenghua Qi, Qingyi Zhu, JunLi Li, Wei Li, Jianjun Tang, Qiming Liu

**Affiliations:** ^1^Department of Cardiovascular Medicine, The Second Xiangya Hospital of Central South University, Changsha, China; ^2^Department of Cardiovascular Surgery, The Second Xiangya Hospital of Central South University, Changsha, China; ^3^Department of Radiology, The Second Xiangya Hospital of Central South University, Changsha, China; ^4^Department of Cardiology, Huaihua Hospital of Traditional Chinese Medicine, Huaihua, China

**Keywords:** light-chain cardiac amyloidosis, CA 125, prognostic predictor, overall survival, biomarkers

## Abstract

**Background and Aims:** Patients with light-chain cardiac amyloidosis (AL-CA) are characterized by high levels of serum carbohydrate antigen 125 (CA 125). However, studies have not explored the correlation between CA 125 and AL-CA. The aim of this study was to explore the clinical implications of an increase in CA 125 in patients with AL-CA.

**Methods and Results:** A total of 95 patients diagnosed with AL-CA at the Second Xiangya Hospital were enrolled in this study. Out of the 95 patients with AL-CA, 57 (60%) patients had elevated serum CA 125 levels. The mean age was 59.7 ± 10.0 years with 44 (77.2%) men in the high serum CA 125 group, and 61.8 ± 9.6 years with 28 (73.7%) men in the normal group. Patients with high CA 125 showed higher rates of polyserositis (79.3% vs. 60.5%, *p* = 0.03), higher levels of hemoglobin (117.4 ± 21.9 g/L vs. 106.08 ± 25.1 g/L, *p* = 0.03), serum potassium (4.11 ± 0.47 mmol/L vs. 3.97 ± 0.40 mmol/L, *p* = 0.049), low-density lipoprotein-cholesterol (3.0 ± 1.6 mmol/L vs. 2.3 ± 1.10 mmol/L, *p* = 0.01), and cardiac troponin T (96.0 pg/mL vs. 91.9 pg/mL, *p* = 0.005). The median overall survival times for patients with high or normal serum CA 125 were 5 and 25 months, respectively (*p* = 0.045). Multivariate Cox hazard analysis showed that treatment without chemotherapy (HR 1.694, 95% CI 1.121–2.562, *p* = 0.012) and CA 125 (HR 1.002, 95% CI 1.000–1.004, *p* = 0.020) was correlated with high all-cause mortality. The time-dependent receiver operating characteristic (t-ROC) curve showed that the prediction accuracy of CA 125 was not inferior to that of cardiac troponin T, N-terminal pro-B-type natriuretic peptide (NT-proBNP), and lactate dehydrogenase (LDH) based on the area under the curve.

**Conclusions:** CA 125 is a novel prognostic predictor. High serum CA 125 values are correlated with low overall survival, and the accuracy of predicting prognosis is similar to that of traditional biomarkers in AL-CA.

## Introduction

Cardiac amyloidosis is a condition of systemic amyloidosis with myocardial involvement. It is caused by the deposition of amyloid proteins derived from misfolded transthyretin or immunoglobulin light-chain in the myocardial interstitium, small vessels, and conduction system. These changes lead to increased ventricular wall thickness, diastolic dysfunction, and arrhythmia. Although more than 30 types of amyloids have been characterized, there are three main types of cardiac amyloidosis, including, acquired monoclonal immunoglobulin light-chain cardiac amyloidosis (AL-CA), wild-type transthyretin amyloidosis (wtTTR-CA), and hereditary transthyretin amyloidosis (hTTR-CA) (1–3). The natural course, treatment, and prognosis of different types of cardiac amyloidosis are different and the diagnosis is performed in late stages and maybe missed (4, 5). Despite the advance in diagnostic and treatment approaches, the exact pathophysiological mechanism of AL-CA has not been elucidated, and the prognosis is extremely poor. Therefore, studies should explore the pathophysiology and clinical aspects of AL-CA.

Previous studies indicated that several biomarkers have a demonstrated diagnostic and/or prognostic value in patients with AL-CA such as cardiac troponin T (6, 7), N-terminal pro-B-type natriuretic peptide (NT-proBNP) (7), D-dimer (8), and lactate dehydrogenase (LDH) (9). However, these biomarkers are easily affected by other conditions such as end-stage liver disease and renal failure. Moreover, the biomarkers staging system cannot accurately stratify the risk of subjects. Therefore, a better prediction biomarker is needed to evaluate the condition of patients and predict the prognosis in clinical practice.

Carbohydrate antigen 125 (CA 125) is a tumor marker associated with ovarian cancer, which is a high-molecular-weight soluble glycoprotein produced by serosal epithelium (10, 11). Increased serum CA 125 levels have also been reported in other malignancies, such as hematological malignant tumors like leukemia and non-Hodgkin's lymphoma, breast and lung cancers, melanoma, and gastrointestinal carcinoma, as well as non-malignant conditions including abdominal surgery, bacterial peritonitis, and tuberculosis (12). Previous studies (13–16) reported that elevated serum CA 125 values are associated with the clinical severity, hemodynamic status, and short-term prognosis of patients with heart failure (HF).

Patients with AL-CA are characterized by a high level of CA125. Currently, the prevalence and implications of increased CA 125 levels in AL-CA are unknown. Therefore, the present study sought to explore the associations between serum CA 125 levels and AL-CA, and systematically evaluated the clinical implications of CA 125 elevation in patients with AL-CA.

## Patients and Methods

**Figure 1:**
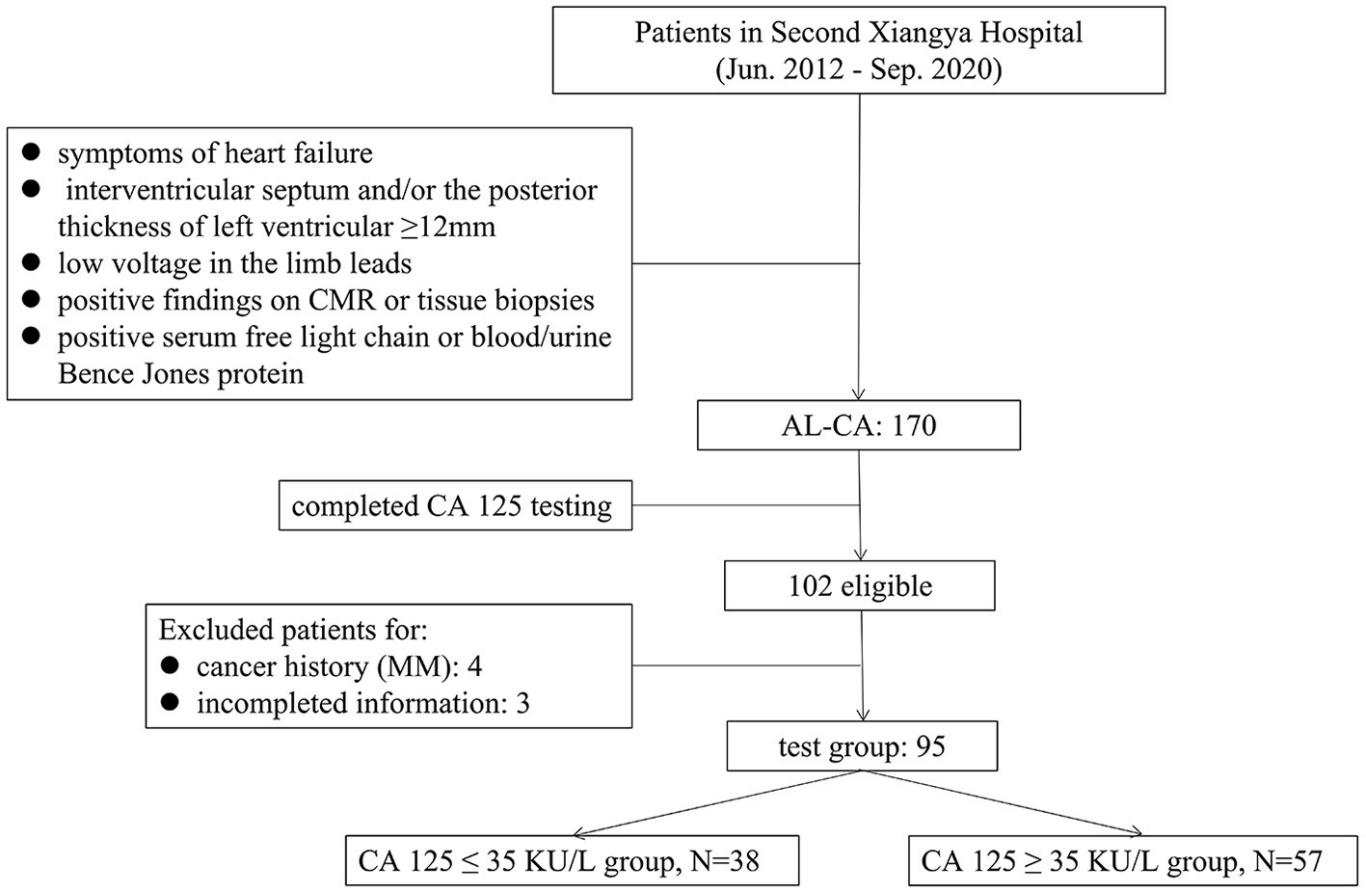
Flowchart for the study population.

A retrospective analysis was conducted on 170 patients diagnosed with AL-CA in the Second Xiangya Hospital of Central South University, from June 2012 to September 2020. The diagnostic criteria for suspected cardiac amyloidosis are symptoms of HF; echocardiography that indicated the interventricular septum and/or the posterior thickness of left ventricular ≥ 12 mm without any other causes of left ventricular hypertrophy; electrocardiogram that showed low voltage in the limb leads; and positive serum free light chain or blood/urine Bence Jones protein. If the suspected criteria are met, cardiac magnetic resonance (CMR) or tissue biopsies will be performed to confirm the diagnosis. The diagnosis of AL-CA was confirmed based on previous literature reports (5) and described as below: (1) positive serum free light chain or blood/urine Bence Jones protein; (2) the presence of apple-green appearance viewed under cross-polarized light with Congo red staining and tissue typing by immunohistochemistry on tissue biopsies from endocardial myocardial tissue or at least one clinically involved organ, including abdominal fat tissue, bone marrow, kidney, and intestinal mucosa; (3) a typical diffuse subendocardial or transmural late gadolinium enhancement pattern on CMR. The compliance of patients with 1+2 or 1+3 was included in this study. The CA 125 test was completed in 102 patients, and four patients with cancer history (except multiple myeloma [MM]), and three patients with incomplete information were excluded. The demographic and clinical characteristics, comorbidities, baseline data of laboratory tests, electrocardiogram and echocardiography data, and treatment of 95 patients with AL-CA were included as the test group (Figure 1). To explore the levels of CA 125 in other diseases, 52 patients with chronic HF (CHF) in the same period were included as one group. AL amyloidosis and MM are plasma cell diseases, and AL amyloidosis is mostly associated with MM (5), therefore, 48 patients who had been diagnosed with MM in the corresponding period were included as another group. Patients with non-cardiac amyloidosis in the two groups, who had a history of cancer diseases and missed CA 125 level data, were excluded. Consequently, the population of the final two groups consisted of 41 and 39 patients.

The study protocol was performed following the ethical guidelines of the Declaration of Helsinki (17). The study was approved by the human research committee of the Second Xiangya Hospital of Central South University.

All patients have undergone venous blood samples for serum levels of CA 125 on the day of admission. Serum CA 125 levels were determined by using electrochemiluminescence (Relia Biotechnology [Jiangsu, China] Co., Ltd), and the cutoff value was set at 35 KU/L.

Follow-up started at the time of diagnosis of AL-CA. The primary endpoint for this study was death from any cause. The survival time (months) was defined as the duration between the diagnosis to the date of death. If the survival time was more than 15 days and <30 days, it would be calculated as 1 month. Data were obtained from medical records or from telephone interviews with patients or relatives by four trained physicians. The last date of follow-up was November 16, 2020. Patients were censored if they were still alive at the end of the research period or were lost to follow-up, on which occasion their last clinic visit or correspondence time was used.

Normally distributed parameters were expressed as mean ± SD, whereas non-normally distributed parameters were expressed as median with interquartile range (Q3-Q1). Categorical values were presented as numbers (percentages). Categorical variables were compared either with Chi-squared or Fisher's exact test. Comparison of continuous variables between two independent groups was performed using unpaired Student's *t*-test (if normally distributed) or Mann–Whitney U-test (non-normally distributed variables). One-way ANOVA or Kruskal-Wallis test was used for comparison of more than two groups. Prognostic factors with *p*-values <0.05 after univariate Cox regression analysis were subjected to multivariate regression analysis to determine the independent factors of predicting survival according to the forward likelihood ratio method. The overall survival was evaluated with Kaplan-Meier curves, and the log-rank test was used to assess the significance of differences between groups. Time-dependent receiver operating characteristic (t-ROC) was used to reflect the accuracy of different biomarkers in predicting the overall survival at various time points based on the area under the curve (AUC). All tests were two-tailed and a *p*-value of <0.05 was considered to be statistically significant. Statistical analysis was performed using Statistical Product and Service Solutions (SPSS) 26.0 (IBM Software Inc), Empower Stats 3.0 software, and R (version 3.3.2) software.

## Results

### Baseline and Characteristics of Patients With AL-CA

The characteristics of 95 patients in the test group were presented in Table 1. Out of the 95 patients, 57 (60%) and 38 (40%) patients were placed into high (CA 125 > 35 KU/L) and normal (CA 125 ≤ 35 KU/L) serum CA 125 groups, respectively. The mean age was 59.7 ± 10.0 years with 44 (77.2%) men in the high serum CA 125 group, and 61.8 ± 9.6 years with 28 (73.7%) men in the normal group. Among all patients with elevated CA 125, 14 (24.5%) patients belonged to New York Heart Association (NYHA) class I-II, whereas 43 (75.5%) patients were at class III-IV. Analysis showed no significant difference in New York Heart Association classification compared with the normal serum CA 125 group (*p* = 0.156). Polyserositis was observed in 46 (80.7%) patients with high serum CA 125 group, compared with 20 (52.6%) patients in the normal CA 125 group (*p* = 0.004). Use of aspirin and furosemide was significantly different between normal and high serum CA 125 groups (28.9% vs. 10.5%, *p* = 0.022; 81.6% vs. 94.7%, *p* = 0.041, respectively). Patients with high serum CA 125 were more likely to present with higher median levels of hemoglobin (117.4 ± 21.9 g/L vs. 106.8 ± 25.1 g/L, *p* = 0.032), serum potassium (4.11 ± 0.47 mmol/L vs. 3.97 ± 0.40 mmol/L, *p* = 0.049), low-density lipoprotein-cholesterol (3.0 ± 1.6 mmol/L vs. 2.3 ± 1.1 mmol/L, *p* = 0.031), cardiac troponin T [96.0 pg/mL (83.3–144.8) vs. 91.6 pg/mL (41.7–96.0), *p* = 0.008], and serum CA 125 [165.4 (114.5–265.9) KU/L vs. 17.9 (11.8–27.3) KU/L, *p* < 0.001] compared with patients with normal serum CA 125. Analysis showed no statistically significant difference in the diameter of atriums and ventricles, the thickness of the ventricular wall, and the ejection fraction. Analysis of ECG showed that patients with elevated CA 125 had lower limb voltage compared with normal CA 125 (76.4% vs. 52.6%, *p* = 0.017).

**Table 1:** Characteristics of 95 patients with AL-cardiac amyloidosis.

	**CA 125 ≤ 35 KU/L** ***N* = 38**	**CA 125 > 35 KU/L** ***N* = 57**	***P*-value**
Age, years	61.8 (9.6)	59.7 (10.0)	0.329
Male, n (%)	28 (73.7%)	44 (77.2%)	0.696
SBP, mmHg	119.2 (27.7)	109.4 (23.5)	0.067
DBP, mmHg	73.7 (14.0)	69.8 (13.0)	0.179
**NYHA, n (%)**			0.156
Class I-II	13 (34.2%)	14 (24.5%)	-
Class III-IV	25 (65.8%)	43 (75.5%)	-
**Mayo AL 2004 stage, n (%)**			0.097
I	2 (5.3%)	0 (0.0%)	-
II	13 (34.2%)	14 (24.6%)	-
IIIa	12 (31.6%)	14 (24.6%)	-
IIIb	11 (28.9%)	29 (50.98%)	-
**Comorbidities, n (%)**			
Multiple myeloma	15 (39.5%)	14 (24.6%)	0.122
Hypertension	15 (39.5%)	16 (28.1%)	0.246
Hyperlipidaemia	9 (23.7%)	15 (26.3%)	0.772
Polyserositis	20 (52.6%)	46 (80.7%)	**0.004**
T2DM	4 (10.5%)	9 (15.8%)	0.465
**Medications, n (%)**			
Pacemaker	4 (10.5%)	2 (3.5%)	0.168
Aspirin	11 (28.9%)	6 (10.5%)	**0.022**
Statins	17 (44.7%)	15 (26.3%)	0.063
ACEI or ARB	11 (28.9%)	16 (28.1%)	0.926
Furosemide	31 (81.6%)	54 (94.7%)	**0.041**
Digitalis	7 (18.4%)	12 (21.1%)	0.753
**Chemotherapy regimens, n (%)**			
Thalidomide	18 (47.4%)	16 (28.1%)	**0.055**
Prednisone or dexamethasone	19 (50.0%)	16 (28.1%)	**0.030**
Bortezomib-based	13 (34.2%)	9 (15.8%)	**0.037**
Melphalan-based	1 (2.6%)	4 (7.0%)	0.348
Palliative care, n (%)	18 (47.4%)	39 (68.4%)	**0.043**
Laboratory results			
Hemoglobin, g/L	106.8 (25.1)	117.4 (21.9)	**0.032**
ALB, g/L	29.7 (6.6)	28.5 (7.1)	0.402
LDH, U/L	261.8 (225.7–317.3)	255.3 (216.8–331.5)	0.258
Potassium, mmol/L	3.97 (0.40)	4.11 (0.47)	**0.049**
Calcium, mmol/L	2.1 (0.2)	2.0 (0.2)	0.064
LDL-C, mmol/L	2.3 (1.1)	3.0 (1.6)	**0.031**
TC, mmol/L	3.8 (1.6)	4.5 (2.5)	0.115
CRP, mg/L	18.6 (25.5)	25.6 (36.9)	0.480
ESR, mm/h	57.7 (39.6)	35.8 (30.8)	**0.011**
Cardiac troponin T, pg/mL	91.9 (41.7–96.0)	96.0 (83.3–144.8)	**0.008**
NT-proBNP, pg/mL	5618.6 (3309.0–10309.5)	8987.0 (4970.3–11649.0)	0.164
D-Dimer, ug/mL	1.52 (1.92)	2.13 (2.40)	0.221
CA 125, KU/L	17.9 (11.8–27.3)	165.4 (114.5–265.9)	**< 0.001**
eGFR, mL/(min ×1.73 m^2^)	62.8 (35.5)	58.6 (31.8)	0.567
24-h urine protein ≥ 1.0 g/24 h	26 (68.4%)	36 (63.2%)	0.598
**Echocardiography**			
LVEDd, mm	45.5 (7.2)	43.5 (6.4)	0.160
RVEDd, mm	32.6 (5.7)	32.9 (5.1)	0.560
LAESd, mm	40.6 (8.1)	41.2 (9.0)	0.759
RAESd, mm	37.9 (9.1)	39.3 (10.6)	0.514
IVS, mm	13.2 (3.1)	14.1 (3.8)	0.213
LVPW, mm	12.8 (2.9)	13.6 (3.3)	0.238
LVEF (%)	54.9 (8.1)	53.0 (10.2)	0.338
**Electrocardiogram**			
Atrial fibrillation, n (%)	6 (15.8%)	11 (20.0%)	0.606
Low limb voltage, n (%)	20 (52.6%)	42 (76.4%)	**0.017**
PRWP, n (%)	29 (76.3%)	47 (85.5%)	0.262

*Data are (N) Mean (SD) or (N) n (%), Median (Q3–Q1), where N is the total number of patients with available data.*

*ACEI, Angiotensin-Converting Enzyme Inhibitor; AL-CA, Light-Chain amyloidosis; ALB, Albumin; ARB, Angiotensin Receptor Blocker; ThD/LeD, Thalidomide/Lenalidomide; CA 125, Carbohydrate Antigen 125; T2DM, Type 2 Diabetes Mellitus; ESR, Erythrocyte Sedimentation Rate; CRP, C-Reactive Protein; DBP, Diastolic Blood Pressure; eGFR, estimated Glomerular Filtration Rate; ESR, Erythrocyte Sedimentation Rate; IVS, Interventricular Septum; LAESd, Left Atrium End Systolic diameter; LDH, Lactate Dehydrogenase; LDL-C, Low Density Lipoprotein-Cholesterol; LVEDd, Left Ventricular End Diastolic diameter; LVEF, Left Ventricular Ejection Fraction; LVPW, Left Ventricular Posterior Wall; NT-proBNP, N-terminal pro–B-type Natriuretic Peptide; NYHA, New York Heart Association; PCT, Procalcitoin; PRWP, Poor R-wave progression; RAESd, Right Atrium End Systolic diameter; RVEDd, Right Ventricular End Diastolic diameter; SBP, Systolic Blood Pressure; TC, Total Cholesterol. Bold values are statistical significance p <0.05*.

### Level of Serum CA 125 in Different Groups

**Figure 2:**
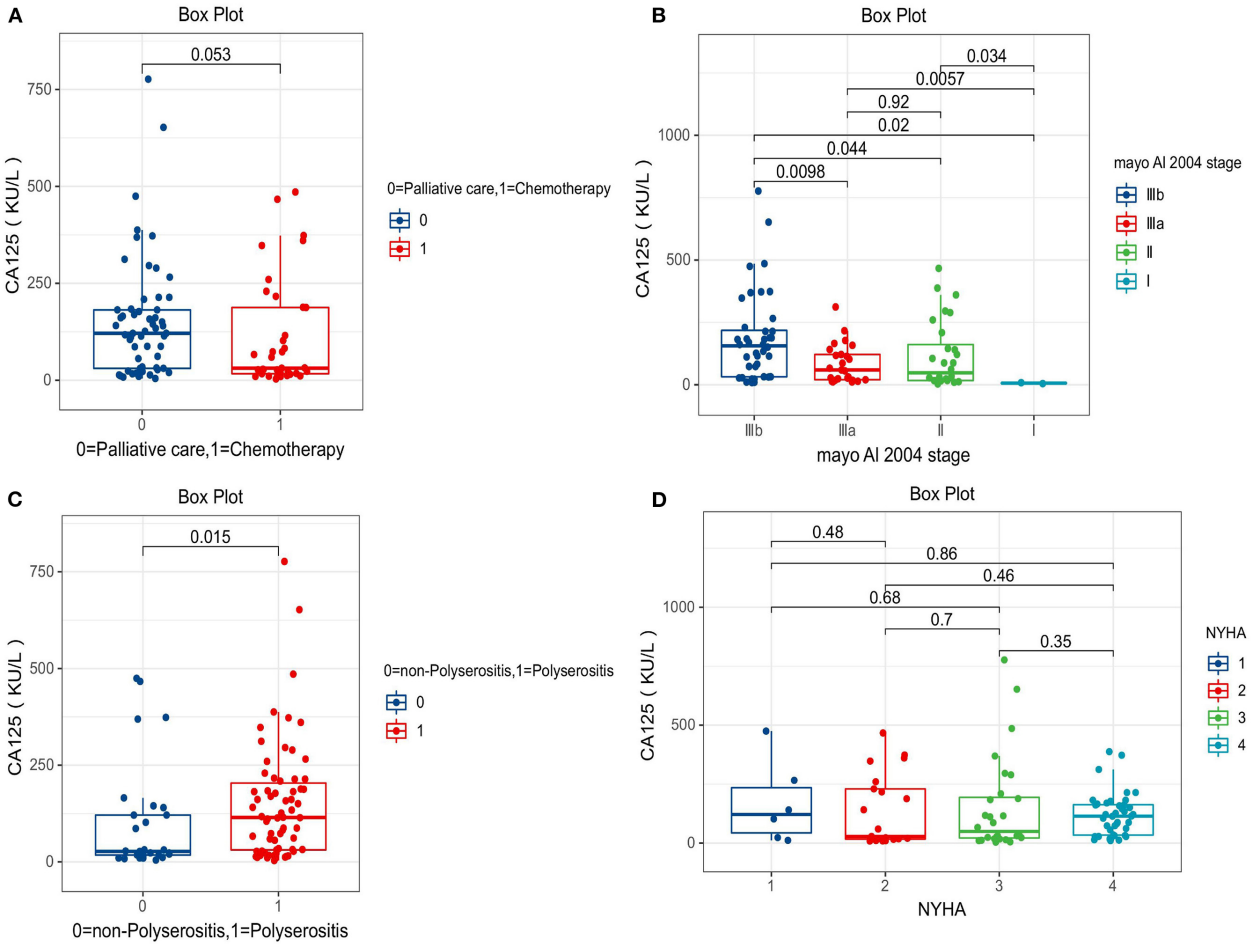
Comparison of serum carbohydrate antigen 125 (CA 125) levels between different groups. **(A)** Palliative care group vs. chemotherapy group (150.8 ± 153.7 vs. 109.9 ± 138.7). **(B)** Mayo AL 2004 stage I (6.5 ± 2.6), II (117.1 ± 134.4), IIIa (84.4 ± 77.7), IIIb (185.3 ± 178.0). **(C)** Polyserositis group vs. non-Polyserositis group (150.2 ± 150.6 vs. 100.0 ± 140.4). **(D)** NHYA I (169.6 ± 175.6), II (134.2 ± 151.5), III (150.6 ± 202.5), IV (119.1 ± 92.2).

The level of serum CA 125 in the polyserositis group was higher compared with that in the non-polyserositis group (150.2 ± 150.6 vs. 100.0 ± 140.4, *p* = 0.015). In the palliative care group, the CA 125 level was higher than the chemotherapy group (150.8 ± 153.7 vs. 109.9 ± 138.7, *p* = 0.053) as well. CA 125 levels varied among different Mayo AL 2004 stages and were statistically different, with higher Mayo stage associated with higher CA 125 levels [I (6.5 ± 2.6), II (117.1 ± 134.4), IIIa (84.4 ± 77.7), IIIb (185.3 ± 178.0)]. Serum CA 125 levels were not statistically different among different NYHA classifications [I (169.6 ± 175.6), II (134.2 ± 151.5), III (150.6 ± 202.5), IV (119.1 ± 92.2); Figure 2].

Further, the levels of CA 125 in different diseases were explored. Clinical characteristics of the test group and the other three groups were shown in Supplementary Table 1. Elevated serum CA 125 values were observed in 57 (60.0%) patients in the AL-CA group, with a mean value of 134.9 ± 148.6 KU/L, in 6 (54.5%) patients in the TTR-CA group, with a mean value of 112.4 ± 134.7 KU/L, in 16 (39.0%) patients in the CHF group with a mean value of 45.7 ± 44.9 KU/L and in 3 (7.7%) patients in the MM group with a mean value of 18.9 ± 20.9 KU/L (*p* < 0.01), Supplementary Figure 1.

### Univariate and Multivariate Predictors of All-Cause Mortality

Prognostic factors for all-cause mortality were explored using univariate and multivariate Cox hazard analyses (Table 2). Univariate analysis showed that systolic blood pressure (HR 0.988, 95% CI 0.976–0.997, *p* = 0.010), diastolic blood pressure (HR 0.981, 95% CI 0.965–0.998, *p* = 0.025), palliative care (HR 2.259, 95% CI 1.309–3.898, *p* = 0.003), low-density lipoprotein-cholesterol (HR 1.232, 95% CI 1.039–1.461, *p* = 0.017), total cholesterol (HR 1.163, 95% CI 1.033–1.310, *p* = 0.013), CA 125 (HR 1.002, 95% CI 1.001-1.004, *p* = 0.001), interventricular septum (HR 1.049, 95% CI 1.000–1.136, *p* = 0.048), and left ventricular posterior wall (HR 1.085, 95% CI 1.004–1.174, *p* = 0.040) were statistically significant predictors of overall survival. However, multivariate analysis showed that the only independent predictors were palliative care (HR 2.613, 95% CI 1.300–5.251, *p* = 0.007) and CA 125 (HR 1.002, 95% CI 1.000–1.004, *p* = 0.033).

**Table 2:** Univariate and multivariate Cox hazard analyses of predictors for all-cause mortality.

**Variables**	**Univariate**			**Multivariate**		
	**HR**	**95% CI**	***p*-value**	**HR**	**95% CI**	***p*-value**
Male	0.797	0.452–1.407	0.435	-	-	-
Age	1.008	0.986–1.031	0.473	-	-	-
NYHA	1.210	0.941–1.580	0.295	-	-	-
SBP	0.988	0.976–0.997	**0.010**	0.988	0.964–1.011	0.302
DBP	0.981	0.965–0.998	**0.025**	0.995	0.953–1.038	0.995
MM	1.352	0.796–2.298	0.265	-	-	-
Hyperlipidemia	1.650	0.931–2.277	0.105	-	-	-
Polyserositis	1.060	0.633–1.776	0.824	-	-	-
Hemoglobin	1.008	0.999–1.018	0.464	-	-	-
ALB	0.973	0.999–1.019	0.078	-	-	-
Calcium	0.987	0.326–2.985	0.981	-	-	-
eGFR	0.998	0.991–1.006	0.672	-	-	-
Palliative care	2.259	1.309–3.898	**0.003**	2.613	1.300–5.251	**0.007**
LDL-C	1.232	1.039–1.461	**0.017**	0.822	0.439–1.539	0.539
TC	1.163	1.033–1.310	**0.013**	1.365	0.894–2.085	0.149
D-Dimer	1.097	0.989–1.216	0.079	-	-	-
CA 125	1.002	1.001–1.004	**0.001**	1.002	1.000–1.004	**0.033**
IVS	1.049	1.000–1.136	**0.048**	1.033	0.834–1.280	0.767
LVPW	1.085	1.004–1.171	**0.040**	1.029	0.804–1.316	0.820
LVEF	0.979	0.958–1.012	0.280	-	-	-
Low voltage	0.681	0.404–1.148	0.150	-	-	-
PRWP	0.687	0.339–1.392	0.297	-	-	-

*HR, hazard ratio; CI, Confidence Interval.*

*For other abbreviations, see Table 1. Bold values are statistical significance p <0.05*.

### Kaplan–Meier Analyses of Overall Survival

**Figure 3:**
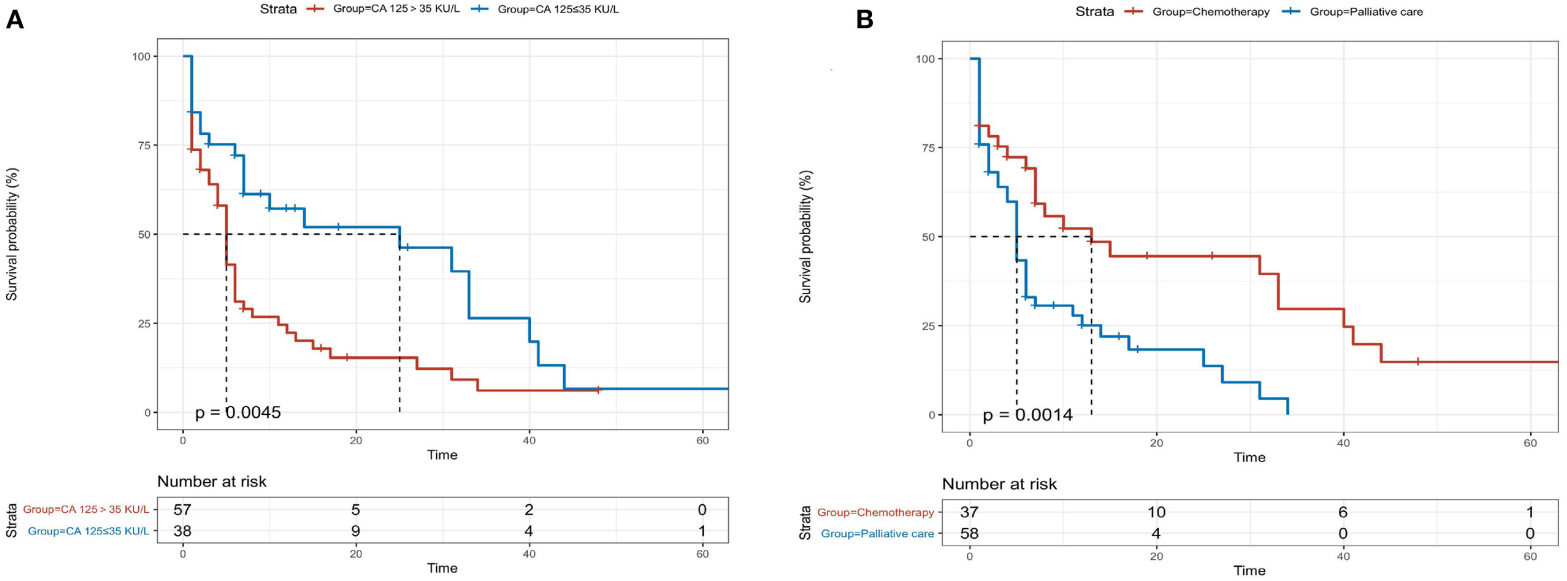
Kaplan-Meier analysis for patients with light-chain cardiac amyloidosis (AL-CA) classified according to serum levels of CA 125 **(A)** and treatment with/without chemotherapy **(B)**. The median overall survival in elevated and normal CA 125 values were 5 months and 25 months, respectively (log rank, *p* = 0.0045). Patients with palliative care had a median overall survival of 5 months while the patients receiving chemotherapy had a median overall survival of 13 months (log-rank, *p* = 0.0014).

Patients with high levels of CA 125 were followed up for a median period of 7 months (IQR 1.0–10.2) and those with normal levels were followed-up for a period of 9 months (IQR 1.5–19.0, *p* = 0.10). Forty-six (80.7%) patients died in elevated CA 125 group and 22 (57.9%) patients died in patients with normal CA 125 (*p* = 0.016) during the follow-up period. The median overall survival in patients with high level CA 125 was 5 months (95% CI 3.881–6.119) and 25 months (95% CI 0.602–39.398) in patients with normal CA 125 levels (*p* = 0.012, Figure 3A). Patients with palliative care had a median overall survival of only 5 months (95% CI 4.823–7.177). The overall survival was significantly shorter for patients with palliative care compared with 13 months (95% CI 2.103–23.897) in patients receiving chemotherapy (*p* = 0.035, Figure 3B).

### Biomarkers for Predicting Overall Survival Using t-ROC Analysis

**Figure 4:**
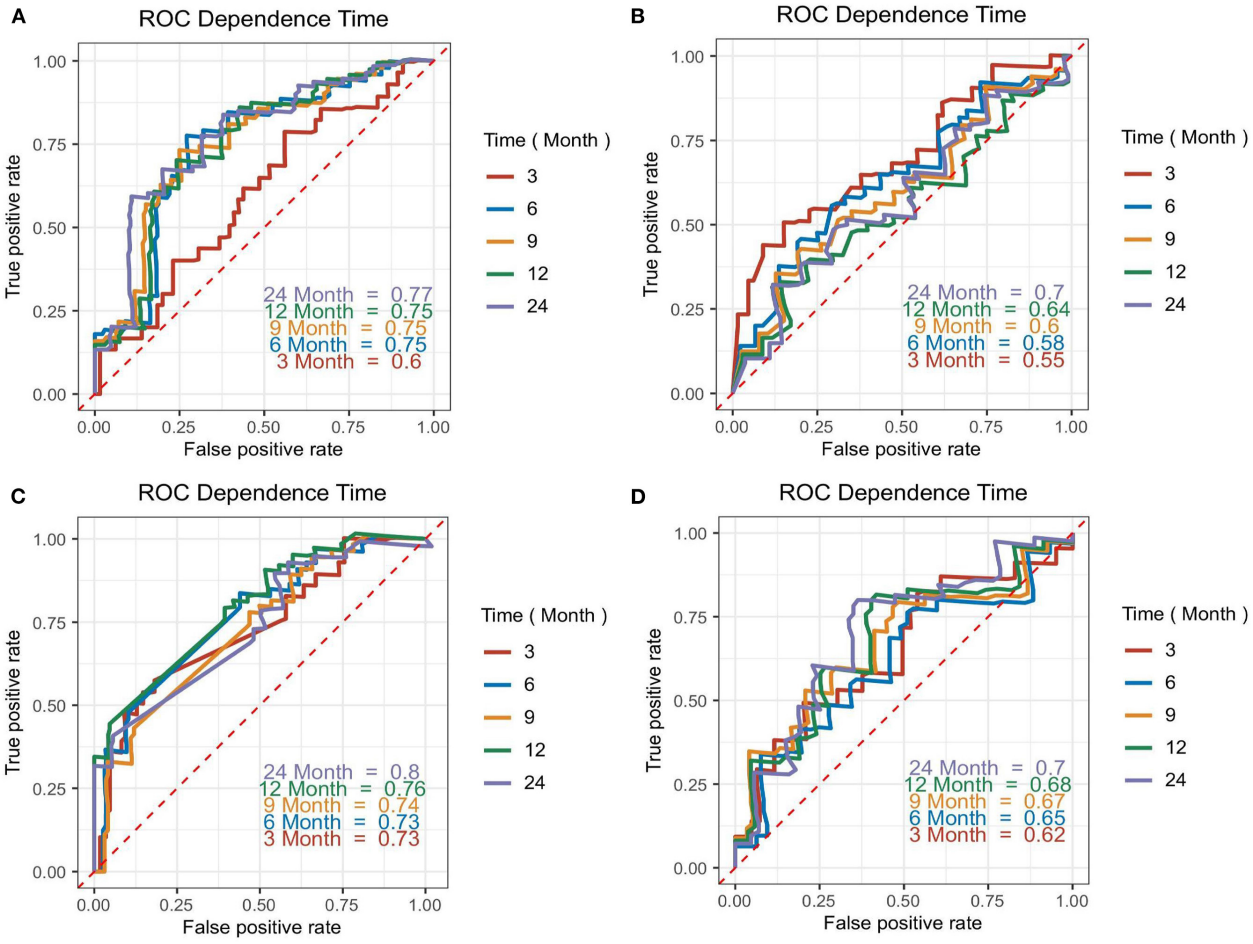
Time-dependent ROC (t-ROC) curves for CA 125 **(A)**, N-terminal pro-B-type natriuretic peptide (NT-proBNP) **(B)**, cardiac troponin T **(C)**, and lactate dehydrogenase (LDH) **(D)** models. The area under the curve (AUC) presented the accuracy of predicting overall survival time (from diagnosis confirmed) for each biomarker at various time points.

The accuracy of the four biomarkers for predicting overall survival was explored by t-ROC analysis. The AUC of 3-months, 6-months, 12-months, and 24-months overall survival for CA 125 were 0.60, 0.75, 0.75, and 0.77, respectively (Figure 4A), compared with NT-proBNP (Figure 4B), cardiac troponin T (Figure 4C), and LDH (Figure 4D).

## Discussions

AL-CA is the most common type of infiltrative cardiomyopathy. It is characterized by various clinical manifestations such as congestive HF, arrhythmia, orthostatic hypotension, syncope, or even other system expressions like gastrointestinal symptoms, albuminuria, and carpal tunnel syndrome leading to a high rate of missed diagnosis (1). In the present study, we analyzed serum CA 125 levels in 95 consecutive patients with AL-CA at an expertise center in China. To the best of our knowledge, this is the first study to explore the prevalence and evaluate the clinical significance of increased CA 125 levels in patients with AL-CA. The principal results of this study were as follows: (1) serum CA 125 levels were elevated in more than half of patients with AL-CA, compared with those with normal CA 125 levels. Patients who exhibited high serum CA 125 showed higher levels of hemoglobin, LDL-C, TC, and cardiac troponin T, and showed higher rates of polyserositis and low limb voltage compared with those with normal levels of CA 125; (2) patients with polyserositis or those who treated with palliative care seemed to express higher CA 125 levels in AL-CA, and higher Mayo stage was associated with higher CA 125 levels; (3) the values of CA 125 in patients with AL-CA were significantly higher compared with those in patients with CHF and MM; (4) CA 125 was a significant independent predictor of survival, with higher levels independently correlated with lower overall survival; (5) the prediction accuracy of CA 125 was not inferior to that of cardiac troponin T, NT-proBNP, and LDH based on the AUC. In addition, CA 125 seemed to be not affected by the estimated Glomerular Filtration Rate (eGFR) status of patients.

Serum CA 125 values are used for diagnosis and follow-up of patients with ovarian cancer and to evaluate the response to therapy (18, 19). An increase in serum CA 125 has also been observed in other malignancies (20–24) and non-malignant diseases (25–28). The first study on the relationship between CA 125 and the cardiovascular system investigated the association between serum CA 125 levels and pericardial effusion in 1993 (29). Following this, Nägele et al. (16) first revealed that CA 125 may be a valuable tool for monitoring the status and clinical course of patients with HF. A previous study (15) demonstrated that only CA 125 levels were correlated with baseline clinical status in CHF compared with CA 19-9, CA 15-3, carcinoembryonic antigen (CEA), and alpha-fetoprotein (AFP), and the serum CA 125 levels of patients with CHF were significantly higher in NYHA class III/IV compared with those in NYHA class I/II. In addition to CHF, elevated CA 125 values were observed in acute HF (AHF) and have been used to assess 6-months risk stratification in patients admitted with AHF (30). Studies have confirmed that cardiac troponin T and NT-proBNP have significant clinical values in determining the prognosis for newly diagnosed patients with AL-CA. Therefore, the Mayo Clinic established a staging system using the two biomarkers (as well as free light chain) to predict patient outcomes (31, 32). This risk stratification system is also the most commonly used in clinical practice. However, the Mayo AL-stage is tremendously affected by renal function. The values of troponin and NT-proBNP in patients with decreased eGFR are severely overestimated, which leads to the conclusion that this system does not truly reflect the prognosis of patients with AL-CA, and novel prognostic biomarkers need to be continuously explored in clinical practice.

The potential mechanism of serum CA 125 levels elevation in AL-CA remains unclear. Seo et al. (29) reported that among 57 patients with different etiologies of pericardial effusion, 65% of the patients had significantly higher serum CA 125 levels compared with normal patients. Moreover, the levels of CA 125 decreased or normalized with the reduction or disappearance of effusion. In addition, the study used anti-CA 125 antibodies to stain the pericardial tissue obtained through autopsy of 17 patients and showed higher serum CA 125 levels in the CA 125-positive-stained pericardium compared with those in negative-stained for CA 125. Except for pericardial effusion, elevated serum CA 25 levels have been reported in pleural and peritoneal effusions with non-malignant diseases and reported that CA 125 may be produced from the mesothelial cells of pleura and peritoneum (33–35). These findings were consistent with findings from our study that polyserositis was observed in 80.7% of 57 patients with AL-CA with elevated CA 125 levels, and we also found that patients with polyserositis were more likely to show higher CA 125 values (*p* = 0.015). Studies reported that blood levels of cytokines and/or their receptors, including Interleukin (IL)-6, IL-10, and tumor necrosis factor (TNF)-α, were more likely to be increased in patients with HF, and cytokine network activation is one of the main factors for serum CA 125 elevation in patients with CHF dependent on inflammation (36–38). Serum CA 125 levels with AL-CA were significantly higher in our study compared with those of patients with CHF (*p* < 0.01), although the blood mean levels of CA 125 in CHF were above normal. Analysis showed that only three patients with MM had a mild elevated serum CA 125 levels (*p* < 0.01). Therefore, the effect of plasma cell diseases on CA 125 was excluded. Previous findings and findings from the current study showed that the reasons for the elevation of serum CA 125 levels in AL-CA may be as follows: (1) abnormal deposition of amyloid in the serosal tissue and increased chronic right ventricular filling pressure caused by CHF leading to tissue stretching and stimulation of secretion by mesothelial cells; (2) amyloid activates the cytokine network by inflammation excitation and stimulates the mesothelial cells to produce and secrete CA 125. However, the underlying mechanism linked between CA 125, cytokines, CA 125-producing cells, and AL-CA should be explored further.

Our study revealed that the levels of cardiac troponin T were associated with the serum CA 125 levels. Higher cardiac troponin T levels were observed in the high serum CA 125 group compared with the level in the normal CA 125 group. Patients with elevated serum CA 125 may have more fluid accumulation leading to increased left ventricular filling pressure and polyserositis, thus causing increased wall stress due to diastolic dysfunction and compressed myocardial capillaries, inducing deficient myocardial blood supply and myocardial ischemia (6). In addition, patients with high serum CA 125 values had higher levels of hemoglobin, potassium, LDL-C, and TC. These findings imply that in most of the patients with AL-CA with elevated CA 125 levels, the kidney is involved, and combined with nephrotic syndrome (39), resulting in entry of body fluid to the interstitial space or serous cavity, leading to blood concentration and dyslipidemia. The significant difference between the serum CA 125 levels of low limb voltage can be attributed to serous effusion. However, the pathophysiological mechanisms should be explored in subsequent studies.

CA 125 was significantly correlated with prognosis after adjusting for systolic blood pressure, diastolic blood pressure, treatment without chemotherapy, low-density lipoprotein-cholesterol, total cholesterol, interventricular septum, and left ventricular posterior wall. The median overall survival of high levels of patients with CA 125 was only 5 months, whereas the median overall survival in normal CA 125 levels was 25 months. This finding implies that high levels of serum CA 125 are independently correlated with high mortality in patients with AL-CA. The accuracy of CA 125 in predicting the overall survival was not inferior compared with the classical prognostic biomarkers including cardiac troponin T, NT-proBNP, and LDH. The possible advantages of CA 125 compared with these biomarkers include being easy to obtain, repeatable, no preparations required, and inexpensive cost (<4 dollars per determination in China compared with more than 40 dollars for NT-proBNP). Notably, CA 125-guided therapy (keeping CA 125 levels at 35 KU/L or less by optimizing the use of a diuretic, enforcing the use of statins, and increasing the frequency of monitoring visits) is superior compared with the standard of care for AHF by reducing the risk of 1-year death and the rate of rehospitalization (40). Further studies should adjust the treatment strategy for patients with AL-CA to reduce the myocardial injury, improve the clinical condition of patients, assist chemotherapy, and decrease the rate of mortality and readmission based on serum CA 125 values.

## Limitations

Several limitations of the study need to be addressed. First, the small sample size and information bias may affect the results of our study. Further research should be conducted with a larger sample size and minimize the information bias for more reliable results. Second, the effect of therapies including chemotherapy and palliative care on serum CA 125 levels was not explored, which may have a crucial influence on the evaluation of treatment outcome and short-term prognosis. Third, although analysis showed no significant difference in eGFR between different serum CA 125 levels groups, CA 125 levels were not evaluated in different renal function stages of patients with AL-CA. Notably, a nephrotic syndrome caused by renal involvement of AL-CA leads to fluid retention and polyserositis and may have caused increased serum CA 125. Therefore, further studies should explore the relationship between CA 125 and nephrotic syndrome.

## Conclusion

The prevalence of elevated serum CA 125 levels is more than 50% in patients with AL-CA. CA 125 is a novel independent prognostic predictor. High serum CA 125 values are correlated with low overall survival and the accuracy of predicting prognosis was not inferior compared with conventional biomarkers.

## Data Availability Statement

The raw data supporting the conclusions of this article will be made available by the authors, without undue reservation.

## Ethics Statement

The studies involving human participants were reviewed and approved by the Human Research Committee of the Second Xiangya Hospital of Central South University. The patients/participants or their legal guardian/next of kin provided their written informed consent to participate in this study.

## Author Contributions

QLiu and JT designed this study and performed quality control of data authenticity. ML drafted the manuscript. ZW and ML collected and analyzed these data. ML, ZW, IT, and NQ performed follow-up visits. JLi, WL, and QZ provided study guidance and revised the paper. QLin, JLiu, NL, YW, MC, YC, QLiu, and JT revised the paper and all authors approved the final version.

## Funding

Financial support was obtained from the National Natural Science Foundation of China (Nos. 81770337 and 81800302), China International Medical Foundation (Z-2016-23-2001-14), and Provincial Natural Science Foundation of Hunan (No. 2019JJ50871).

## Conflict of Interest

The authors declare that the research was conducted in the absence of any commercial or financial relationships that could be construed as a potential conflict of interest.

## Publisher's Note

All claims expressed in this article are solely those of the authors and do not necessarily represent those of their affiliated organizations, or those of the publisher, the editors and the reviewers. Any product that may be evaluated in this article, or claim that may be made by its manufacturer, is not guaranteed or endorsed by the publisher.

## Supplementary Material

Supplementary Figure 1 Comparison of serum CA 125 levels between different groups. CHF, Chronic Heart Failure; AL-CA, Light-chain Cardiac Amyloidosis; TTR-CA, Transthyretin Amyloidosis; CA 125, Carbohydrate Antigen 125; MM, Multiple Myeloma.

Supplementary Table 1 Clinical characteristics of several control groups.
