# Supplementary figures and images for: High Serum Carbohydrate Antigen (CA) 125 Level Is Associated With Poor Prognosis in Patients With Light-Chain Cardiac Amyloidosis
Source: Front Cardiovasc Med. 2021 Oct 28;8:692083. doi: 10.3389/fcvm.2021.692083 (PMC8581134; doi:10.3389/fcvm.2021.692083)

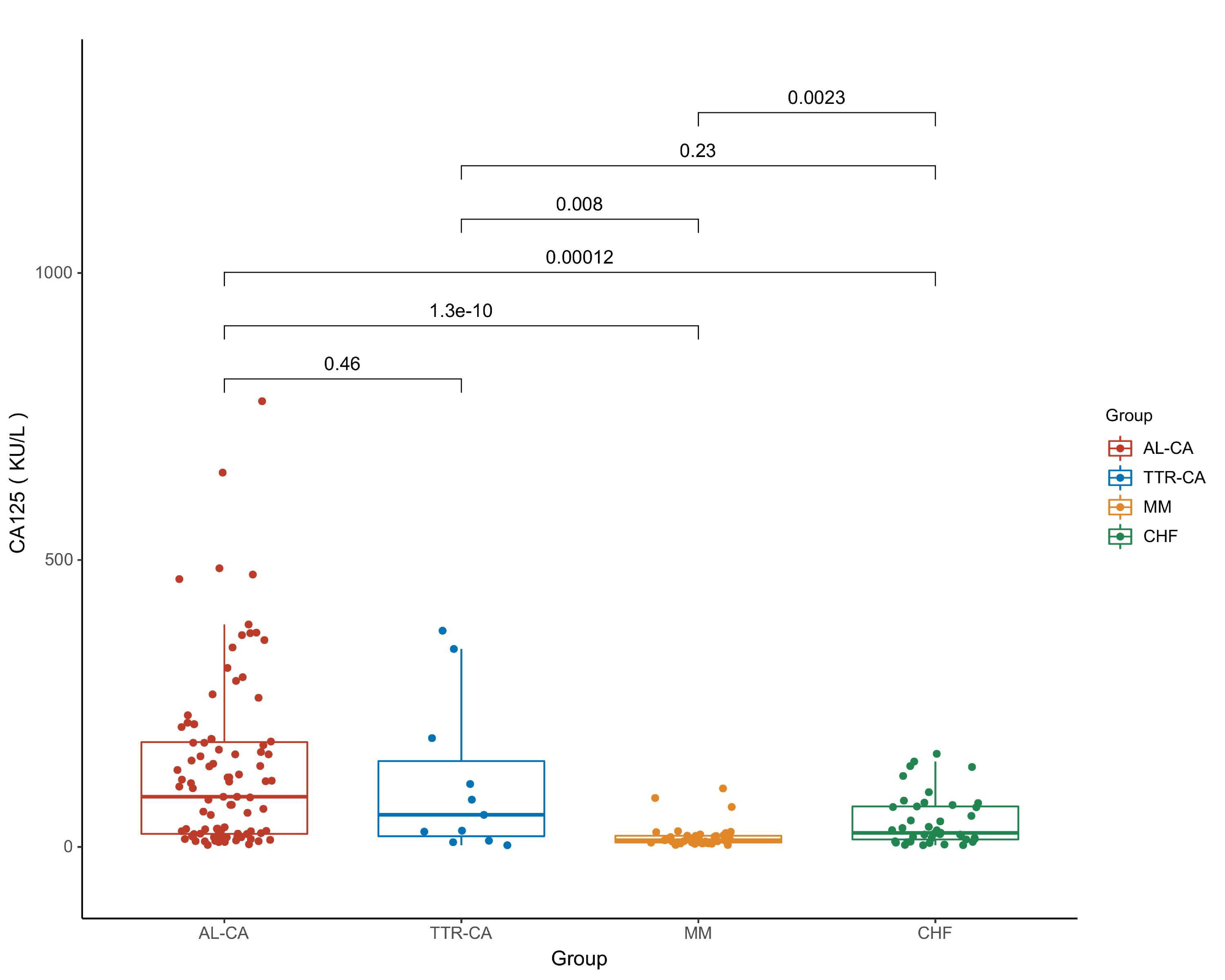

Supplement: Supplementary Figure 1 — Comparison of serum CA 125 levels between different groups. CHF, Chronic Heart Failure; AL-CA, Light-chain Cardiac Amyloidosis; TTR-CA, Transthyretin Amyloidosis; CA 125, Carbohydrate Antigen 125; MM, Multiple Myeloma. [file Image_1.jpeg]
