# Supplementary material for: High Serum Carbohydrate Antigen (CA) 125 Level Is Associated With Poor Prognosis in Patients With Light-Chain Cardiac Amyloidosis
Source: Front Cardiovasc Med. 2021 Oct 28;8:692083. doi: 10.3389/fcvm.2021.692083 (PMC8581134; doi:10.3389/fcvm.2021.692083)
Supplement: Supplementary Table 1 — Clinical characteristics of several control groups. [file Table_1.docx]

**Supplementary Table 1.** Clinical characteristics of several control groups.

|  | AL-CA | TTR-CA | CHF | MM | *p* |
| --- | --- | --- | --- | --- | --- |
|  | (n=95) | (n=11) | (n=41) | (n=39) | Value |
| Age, years | 60.5 (9.9) | 68.4 (13.2) | 72.1 (13.4) | 61.5 (10.9) | **< 0.01** |
| Male, n (%) | 72 (75.8%) | 6 (54.5%) | 24 (58.5%) | 23 (59.0%) | 0.14 |
| NYHA, n (%) |  |  |  |  | - |
| Class Ⅰ | 6 (6.3%) | 0 (%) | 0 (%) | 0 (%) |  |
| Class Ⅱ | 21 (22.1%) | 1 (9.1%) | 0 (%) | 0 (%) |  |
| Class Ⅲ | 28 (29.5%) | 7 (63.6%) | 11 (26.8%) | 0 (%) |  |
| Class Ⅳ | 40 (42.1%) | 3 (27.3%) | 30 (73.2%) | 0 (%) |  |
| Cardiac troponin T, pg/mL | 96.0 (66.0-123.2) | 51.4  (37.6-64.5) | 51.4 (27.9-123.1) | - | 0.20 |
| NT-proBNP, pg/mL | 8087.0 (4033.5-11223.0) | 2825.5 (2645.8-4751.7) | 11423 (4738.0-28715) | - | **< 0.01** |
| CA 125>35 KU/L, n (%) | 57 (60.0%) | 6 (54.5%) | 16 (39.0%) | 3 (7.7%) | **< 0.01** |
| CA 125, KU/L | 134.9 (148.6) | 112.4 (134.7) | 45.7 (44.9) | 18.9 (20.9) | **< 0.01** |

Data are (N) Mean (SD) or (N) n (%), Median (Q3-Q1), where N is the total number of patients with available data.

CHF: Chronic decompensated heart failure; AL-CA: Light-chain cardiac amyloidosis; CA 125: Carbohydrate Antigen 125; MM: mutiple myeloma; NT-proBNP: N-terminal pro–B-type Natriuretic Peptide; NYHA: New York Heart Association; TTR-CA: Transthyretin cardiac amyloidosis
